# A Qualitative Analysis of Patient Perspectives and Preferences in Lupus Management to Guide Lupus Guidelines Development

**DOI:** 10.1002/acr.25693

**Published:** 2025-11-03

**Authors:** Shivani Garg, Izzy Hartel, Lisa R. Sammaritano, Anca Askanase, Bonnie L. Bermas, Maria Dall'Era, Alí Duarte‐García, Victoria P. Werth, Brad Rovin, Reem A. Mustafa, Amy S. Turner, Bené Williams, Brian L. Ung, Hiya Bhavsar, Monique C. Gore‐Massy, Nahirannette Pulido, Natacha Guerrero, Natalie M. Smith, Wambui Machua, Linda T. Hiraki, Mary Beth Son

**Affiliations:** ^1^ University of Wisconsin School of Medicine and Public Health Madison; ^2^ Hospital for Special Surgery, Weill Cornell Medicine New York New York; ^3^ Columbia University New York New York; ^4^ UT Southwestern Medical Center Dallas Texas; ^5^ University of California San Francisco; ^6^ Mayo Clinic Rochester Minnesota; ^7^ University of Pennsylvania and Corporal Michael J. Crescenz Veterans Affairs Medical Center Philadelphia; ^8^ Ohio State University Columbus; ^9^ University of Kansas Kansas City; ^10^ American College of Rheumatology Atlanta Georgia; ^11^ Patient Advisory Panel Members; ^12^ The Hospital for Sick Children Toronto Ontario Canada; ^13^ Boston Children's Hospital Boston Massachusetts

## Abstract

**Objective:**

A patient‐centered approach for chronic disease management, including systemic lupus erythematosus (SLE), aligns treatment with patients’ values and preferences, leading to improved outcomes. This paper summarizes how patient experiences, perspectives, and priorities informed the American College of Rheumatology (ACR) 2024 Lupus Nephritis (LN) and 2025 SLE screening, treatment guidelines.

**Methods:**

We completed a cross‐sectional qualitative study using content analysis of two Patient Panel meetings for the ACR LN and SLE guidelines. Key themes were presented by Patient Panel representatives during Voting Panel Meetings along with evidence for each recommendation, to ensure comprehensive discussions and align treatment recommendations with patients’ priorities and values.

**Results:**

Nineteen people (90% women) with diagnoses of SLE and/or LN participated in the Patient Panels and 17 consented to use their feedback for analysis. Thematic analysis of their discussions revealed nine patient‐reported key themes in three domains: (1) treatment and monitoring of LN and SLE: medication side effects, daily function, treatment goals, and monitoring and screening; (2) clinical communication: strategies to optimize communication and provider and structural impediments to effective communication; and (3) improving transparency and information sharing: clinical trial participation, and medical costs and insurance coverage. These themes were actively incorporated into discussions during the Voting Panels for the ACR LN and SLE guidelines.

**Conclusion:**

This work supported the integration of patient experiences in the clinical practice guideline development process and aligned recommendations with real‐world patient experiences and priorities, thereby enhancing the clinical applicability of the ACR LN and SLE guidelines.

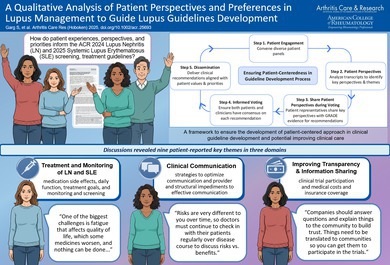

## Graphical abstract

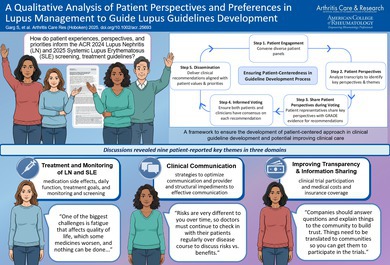

## INTRODUCTION

Systemic lupus erythematosus (SLE) is a life‐threatening autoimmune disease, with lupus nephritis (LN) as one severe manifestation.^1^, ^2^, ^3^, ^4^, ^5^, ^6^, ^7^, ^8^, ^9^, ^10^, ^11^, ^12^, ^13^, ^14^, ^15^ SLE and LN are chronic diseases characterized by disease flares, heterogeneity in disease severity, and significant impact on quality of life.^1^, ^2^, ^3^, ^4^, ^5^, ^6^, ^7^, ^8^, ^9^, ^10^, ^11^, ^12^, ^13^, ^14^, ^15^ Treatment is commonly aimed at controlling the disease and minimizing target organ damage. However, there is discordance between disease activity indices and patient reported outcomes, underscoring the importance of aligning treatment with patients’ values and preferences.^16^, ^17^, ^18^, ^19^, ^20^, ^21^ Incorporating patient perspectives in clinical practice guidelines can assist in balancing benefits with potential harms of treatments as well as include patients’ values and preferences.^22^, ^23^, ^24^ This approach acknowledges that the lived experiences of individuals with SLE extend beyond standardized measures and encompass a spectrum of physical, emotional, and social challenges, and support a patient‐centric treatment plan. Finally, including patient voices in clinical guidelines improves feasibility and acceptability of using guideline recommendations during routine visits.^25^

**SIGNIFICANCE & INNOVATIONS**

- This paper summarizes key themes of patient experiences, perspectives, and priorities to support shared decision‐making and align treatment recommendations in lupus nephritis and systemic lupus erythematosus guideline development.
- Three thematic domains emerged: treatment and monitoring; clinical communication; and improving transparency and information sharing.
- We highlight strategies to engage patients with diverse experiences to inform guideline recommendations per patient perspectives.

Since 2015, the American College of Rheumatology (ACR) has prioritized formal patient involvement in guideline development.^24^, ^26^ Clinical guideline development experts have highlighted the need to ensure patients’ values are taken into consideration when evidence is systematically evaluated and graded to make recommendations. The goals of patient engagement in this setting are to: 1) identify patient values and priorities to inform clinical practice recommendations, particularly about therapies that carry potential harms; and 2) foster a collaborative approach to care, enhance patient engagement and adherence, and provide a more holistic and patient‐centered approach for chronic disease care.

To inform the 2024 and 2025 ACR guidelines for the screening, monitoring, and treatment of LN and SLE respectively, the ACR convened two Patient Panels. The objectives of this manuscript are to highlight key themes that emerged during the Patient Panel discussions and illustrate how patient engagement can be actively incorporated into guideline development.

## MATERIALS AND METHODS

### Study design

We completed a cross‐sectional qualitative study using content analysis of Patient Panel meetings that were held to inform ACR LN and SLE guideline development. This approach has been successfully used in ACR interstitial lung disease (ILD) and other guidelines to identify complex latent constructs that are not easily measured.^24^, ^26^ The consolidated Criteria for Reporting Qualitative Research was used to design this study (Supplementary Table 1).^28^, ^29^

### Setting and participants

National surveys, recommendations from the ACR SLE and LN guideline committee members, communications through the Lupus Foundation of America patient support groups, and previous Patient Panel member lists were used to identify participants for the Patient Panel meetings. Patients completed a screening checklist to report their diagnosis, disease type (SLE or LN), disease control (good or partial control with or without immunosuppressive medicines), rheumatologist, region, and ethnic group. All patients (n = 28) who reported a diagnosis of SLE with disease type as SLE or LN regardless of good or partial disease control and not actively followed by the clinician facilitators leading the Patient Panel meetings were invited to participate in the Patient Panels. Among all invited patients, 15 individuals participated in LN panel meeting and 13 participated in the SLE panel meeting; 9 individuals participated in both LN and SLE Patient Panel meetings.^27^ In total 19 unique individuals participated in at least one Patient Panel meeting and 17 out of 19 consented for using their feedback to develop this manuscript. Sample size selection and the number of focus groups were determined based on prior guidelines, feedback from the guideline leadership (Core Team), and expert facilitators. Participants for the Patient Panels were purposefully recruited to represent different cultural, social and ethnic backgrounds, disease duration, and age of onset to capture different patient perspectives and values and to ensure that the findings were generalizable and applicable to different populations with lupus. Two separate Patient Panels, one for LN and one for SLE, were convened to identify key themes on patient experiences, values, perceptions, and priorities.

### Patient panel meetings

Three Core Team rheumatologists (SG, LTH, MBS), supported by two ACR staff members experienced with ACR guideline development processes (AST and RP), facilitated the two four‐hour virtual Patient Panel meetings. Prior to the virtual meetings, patients received a synopsis of the evidence that had been compiled to create each guideline. Additionally, the facilitators created and shared a semi‐structured outline of the topics covered by the recommendations. Lastly, patients participated in an orientation webinar that provided information and guidance on interpreting the evidence report before the Patient Panel meeting. Participants were encouraged to consider the recommendations in the context of their preferences, values and priorities in regard to monitoring and management of SLE or LN. A facilitated discussion started with open‐ended, guiding questions (Supplementary File 1). The facilitators provided definitions and gave details whenever necessary. Questions and prompts were kept open‐ended by the facilitators to expand discussion points and engage panel members. To reduce participant fatigue during the four‐hour Patient Panel meetings, facilitators incorporated scheduled breaks (every 60 minutes) and participants were encouraged to take additional breaks as needed. Facilitators also monitored engagement and ensured equal participation throughout the meetings.

The Patient Panel meetings were recorded by the ACR team (AST and RP), and quotes and comments made by Patient Panel members were documented. Text transcripts were developed from the recordings and discussions. All identifiers and identifiable comments were redacted from the text transcripts. Text transcripts were independently reviewed by the facilitators for accuracy and to achieve immersion. Key themes were noted by each facilitator, and discrepancies in coding and generated themes were resolved via discussion to enhance trustworthiness and rigor. Key themes were reviewed with the Patient Panel members to obtain feedback on the findings. Given this was a secondary use of de‐identified transcripts originally generated for the ACR guidelines development work, an institutional review board approval was not required.

### Voting Panel meetings

The ACR uses the Grading of Recommendations Assessment, Development and Evaluation (GRADE) framework during guideline development. GRADE provided a structured framework for rating the quality of evidence and determining the strength of recommendations based on a careful assessment of benefits, harms, and patient perspectives. The published literature was reviewed by the Literature Review Team, which also designated the quality of evidence for each recommendation, per GRADE. This evidence was then presented during the Voting Panel meetings by the meeting chair and Literature Review Team leader. The Voting Panel meetings occurred virtually over two days for LN and over three days for SLE. Two Patient Panel members were selected to be patient representatives on the Voting Panels for the 2025 ACR SLE and 2024 LN Guidelines to highlight and share perspectives and key themes that emerged from the Patient Panel meetings around each recommendation and/or topic discussed.

During the Voting Panel meetings, the chair presented data detailing the quality of evidence for each recommendation and started the discussion by inviting patient representatives to share their comments. Following detailed discussion, Voting Panel members, both patients and health care providers, voted on each recommendation.

### Content analysis

Transcripts from Patient Panel meetings were analyzed using content analysis. Key themes informed the coding scheme for the content analysis. Content analysis was performed by two independent reviewers (including 1 facilitator, SG, and 1 non‐facilitator, IH).^28^, ^30^, ^31^, ^32^ The data were organized into meaningful thematic clusters pertaining to patient preferences. Adjustments to the coding scheme were made iteratively between each reading until thematic saturation was reached.^26^, ^28^, ^30^, ^31^, ^32^ After key themes were generated, the reviewers and facilitators met to discuss themes and coding. Using validated qualitative methods, any discrepancies in coding and generated themes were resolved via discussion and consensus to enhance trustworthiness and rigor. Transcripts were coded using NVivo software.^33^ This research triangulation was employed to enhance credibility of the findings and ensure that the analysis reflects the full breadth and depth of the data. Using member checking, we assured that patient preferences were associated with placement in the respective themes and code categories. The listed frequencies of each coded theme and subcategories within each theme for patient preferences and priorities regarding SLE and LN monitoring and treatment were summarized in tables. Following this, themes and subcategories under each theme were ranked in descending order of the listed frequencies. This step helped us identify highly ranked or high priority themes and subcategories within each theme. Finally, highly ranked themes with subcategories within each theme (informed by their listed frequency) were summarized in final tables included in this manuscript.

## RESULTS

### Patient panel member characteristics

Among 19 unique individuals participated (attended) in one or two Patient Panels, 17 patients provided consent to use their feedback for analysis and inclusion in aggregate data for this paper; 88% were female, 55% were Black and 77% had LN (Table 1). Regarding representation across US geographic regions, 47% of the Patient Panel members were from the South, followed by 35% from the East.

**Table 1:** Summary of patient panel characteristics

Characteristics	Patients (n = 17)^a^, n (%)
Age, median (range), in yrs	38 (22–50)
Female	15 (88.2)
Race/Ethnicity	
Black	9 (52.9)
Asian	3 (17.6)
White	3 (17.6)
Hispanic	2 (11.8)
Region	
South	8 (47.1)
East	6 (35.3)
West	3 (17.6)
Disease control^b^	
Good control with 1 or more immunosuppressive medicines^b^	7 (41.2)
Partial control requiring multiple immunosuppressive medicines^c^	10 (58.8)
Disease experience	
Arthritis	16 (94.1)
Skin manifestations	13 (76.5)
Lupus nephritis diagnosis	14 (82.3)
Lupus affecting blood cell counts	11 (64.7)
Ever requiring hospitalization for lupus flare	9 (52.9)
Serositis	5 (29.4)
Childhood‐onset lupus	1 (5.88)

^a^ Table shows data from 17 patients who provided consent to use their feedback for analysis for this manuscript among 19 unique individuals who attended 1 or more patient panel meetings.

^b^ Patient‐reported information.

^c^ Requiring multiple medicines or failed several medicines for lupus.

### Thematic analysis

Across the two Patient Panels for LN and SLE, four common themes emerged regarding treatment and monitoring of LN and SLE (Domain 1) including medication side effects, daily function, treatment goals, and monitoring and screening (Table 2). Next, two key themes were generated around clinical communication (Domain 2), including strategies to optimize clinical communication and effective, respectful communication (Table 3). Finally, two themes surfaced around improving transparency and information sharing (Domain 3), including clinical trial participation, and medical costs and insurance coverage (Table 4). As described in our methods, common and key themes were identified based on the ranking informed by the listed frequency of each coded themes and subcategories in our content analysis.

**Table 2:** Domain 1: Treatment and monitoring of systemic lupus erythematosus and lupus nephritis*

Themes	Theme subcategories	Illustrative quotes (1–3)
Theme 1: Medication side‐effects	Impact on quality of life	*“One of the biggest challenges is fatigue that affects quality of life, which some medicines worsen, and nothing can be done…”* *“Some side effects are unbearable, some are tolerable…I think the discussion should be personalized based on each patient's preferences and tolerability…”*
	Balancing side effects vs. efficacy	*“Side effects of therapies do affect quality of life, but I am willing to take all of these side effects over kidney failure.”* *“Prednisone can destroy your bones and teeth […] but when I am in a flare, I know prednisone will definitely help me…”* *“Sad we're still having this fertility conversation as I am unable to have children because of the medication [cyclophosphamide] I was given…”* *“Immunosuppressives causing infections has also been a big issue. This should be discussed with patients more regularly.”* *“Hydroxychloroquine messed up my vision, but I am back on it now as I need it.”*
	Pill fatigue	*“I have pill fatigue, sometimes I just can't do it… It [pill burden] is mentally overwhelming resulting in pill fatigue.”* *“Pill burden is a real problem; providers must remember this and prescribe accordingly, so patients have as few pills to take…”* *“At one point, I was on 14 pills… I am a pre‐med student… I just could not do it…”*
Theme 2: Daily function	Maximizing daily function	*“…My goal is to survive and have the best daily functioning as possible.”* *“Quality of life is my #1 wish but in reality, this doesn't seem possible.”* *“Maximum quality of life… I like to be physically active… I miss time with family…”*
	Avoiding flares	*“Avoiding a flare is very important to be able to achieve … normalcy in life.”* *“Prevent disease flare because [flare] leads to extreme joint pain, finger swelling, [and] inability to walk, which affects daily activities.”* *“Each flare has led to a major ‘failure’ [loss of sight, going to hospital], so avoiding the flare is most important to me.”*
	Preventing organ damage	*“Kidney [function] preservation was very important initially and still is today.”* *“Protecting organ function is a priority”* *“… I am willing to take all of these side effects over kidney failure…”*
	Medicine preferences (number, route, and type)	*“Mode of administration should be considered […] [Discuss] what is easier for the patient or preferred by them…”* *“Start with one medicine, then add others, because then you have a better understanding of side effects and response to each one…”* *“Pill burden is a real issue… It affects cost and adherence…”*
Theme 3: Treatment goals	Survival	*“My goal is to survive…”* *“First priority was survival at time of diagnosis because I was in coma – so I just wanted to live.”*
	Aligning treatment with patient values	*“Understand the patient as a whole person instead of just a disease […] Consider … [what] communities a person is in… all affect goals of treatment.”* *“I was a med student trying to go into residency. It was scary to think of the environment I was getting into. There was no discussion about adjusting my steroids to better manage that risk.”*
	Changes based on disease severity	*“Treatment goals depend on the point in the journey, particularly how severe and active the disease is…”* *“At certain levels of illness, I am more willing to accept potential harms, if options are limited…”* *“When you are really sick, your [goals] are different. You can have other conversations when your illness slows down a little.”*
	Changes with age or life phases	*“I would avoid stomach side effects more when I was younger because I was trying to go out and socialize. As I get older, I'm worried about risk of infection, so I would sit at home with stomach issues, if needed…”* *“Fertility is also important, as lupus attacks women in their prime; need to discuss egg preservation as well as other organ damage issues…”* *“My kidney function has gotten a lot better, so now I am more concerned about fertility and cancer risks and considering my quality of life when I am 40 or older”*
	Long‐term outcomes	*“Doctors can help by really talking with patients about options and plans that look ahead and provide hope, especially about stages of treatment to try to achieve longer‐term goals…”* *“In the beginning, you're still learning and not worried as much about longer term outcomes, but they become more important as you go on.”*
Theme 4: Monitoring and screening	Frequency of blood and urine monitoring	*“I watch liver and kidney function carefully. Can't always get organ transplanted.”* *“I don't mind doing blood work every 3 months, especially if labs are more local to me. I don't prefer to drive 2 hours to do a blood draw.”* *“Over the years, I have come to prefer monthly bloodwork, because it educates me to see my blood levels.”*
	Invasive procedures (eg, diagnostic kidney biopsies)	*“Kidney biopsy was the best method to identify [lupus nephritis]…”* *“[I] Had a kidney biopsy and was told that it would hopefully tell that [I] had LN…”* *“I was put under sedation so I did not have much issues [with the kidney biopsy]…”*
	Repeat invasive procedures	*“I would not likely want to do another biopsy because of other possible side effects like bleeding. I would only do it again if … there was a clear reason.”* *“If I need a biopsy again, I will have to be convinced that it is vital…”* *“I was not sedated and it [kidney biopsy] was painful…”*

* Themes arranged from most common to least commonly listed frequency. Only key subcategories with higher listed/coded frequencies for each theme are shown. LN, lupus nephritis.

**Table 3:** Domain 2: Themes regarding clinical communication*

Themes	Theme subcategories	Illustrative quotes (1–3)
Theme 1: Optimizing clinical communication	Shared decision‐making	*“Risks are very different to you over time, so doctors must continue to check in with their patients regularly over disease course to discuss risks vs. benefits.”* *“A clear discussion and understanding between the doctor and patient to understand what is being recommended is recommended.”* *“Every side effect, severe or mild, should be discussed… This helps with decision‐making and being prepared.”*
	Showing empathy and compassion	*“This [compassion] is important for treatment success, overall. The doctor should be genuine and see the whole patient.”* *“Emphasizing importance of compassionate care and understanding that this is a gateway for trust and better outcomes.”* *“That [compassionate care] let me know that I didn't have to stick with a medication because it ‘worked’, helped be more confident about speaking up.”*
	Multidisciplinary or collaborative care team approach	*“My pharmacist also suggested where I could reduce certain medications, all of which was helpful and made things easier to manage.”* *“The best decision depends on the drug sometimes, and the trust the patient has in their doctor… health care team…”* *“[Doctors] should refer the patient to mental health specialists, social worker.”* *“It would be very helpful to have a referral from the rheumatologist to other areas where we are affected, like GI and cardiology.”*
	Personalized approach	*“Hopefully, over time, we can get to personalized lupus care with more precision in the future […] every patient is different…”* *“The doctor should ask what the patient's most important priority for the visit is, to address the limited time and to be sure they get to that issue.”*
	Offer telehealth and enroll in pharmacy mail delivery services	*“Having a virtual appointment option is very helpful […] This has allowed for better adherence…”* *“Virtual appointments mean better control of my disease…”*
	Building trust and rapport	*“Doctors need to emphasize the importance of compassionate care and understand that this is a gateway for trust and better outcomes.”* *“The best decision depends on the drug… and the trust [the] patient has in [the] doctor…”*
Theme 2. Provider and structural impediments to effective communication	Impact on mental health	*“[I] feel hopeless and experience post‐traumatic stress disorder for all we go through with treatments…”* *“Mental health is one of the most severe side‐effects and should be assessed…”* *“Depression is a basic thing for chronic illness; recognize [this] and do not be dismissive.”*
	Judgmental care team	*“Don't be judgmental, but be curious about why they [the patient] may decline certain treatment options.”* *“Please explain [treatments] and be kind in the way the message is delivered – ‘this is how we think it will help you’ rather than treating the patient like a science experiment.”*
	Structural racism and need for cultural competency	*“Understand the patient as a whole person instead of just a disease such as the concept of fertility as a Black woman and a person with lupus…”* *“Medical school doesn't teach interpersonal skills, cultural competence, and intersectionality but should…”* *“Education for patients & providers about this [cultural competency]‐ patients are often dismissed, even more so for people of color & women.”*
	Mistrust in health care team and system	*“… Some rheumatologists are so focused on the numbers that it's like the patient is not even there. This is dismissive, which makes patient feel worse and less open or willing to listen to their doctor.”* *“We [lupus patients] want to know and be assured that we are not disposable human subjects to pharmaceutical companies.”* *“At this stage of my life, thinking about possibly having to go to a facility several times per week gives me a sense of sadness and grief.”*
	Lack of collaborative approach	*“Sometimes doctors will send patients to another specialist to deal with issues that come up; know that the rheumatologist's referral has more impact…”* *“Pharmacists… I wish we had them there all the time… their input is valuable.”*

* Themes arranged from most common to least commonly listed frequency. Only key subcategories for each theme are shown. GI, gastrointestinal.

**Table 4:** Domain 3: Improving transparency and information sharing*

Theme 1: Clinical trial participation	Building trust among communities	*“Companies should answer questions and explain things to the community to build trust. Things need to be translated to communities so you can get them to participate in the trials.”* *“I mistrust clinical trials because of the history within the Black community. When you ask, the doctor or company say they don't know how many Black women have participated in the trials. People asking people to be involved [in clinical trials] need to know the details.”* *“The rheumatologists who are doing clinical trials don't always tell all of their patients that they are doing the trials […] all potential research opportunities should be provided to all patients.”*
	Additional details on medicines, inclusion criteria, placebo	*“Also, when involved in a clinical trial, the patient doesn't know if they are receiving the drug or a placebo and could get pulled off the trial mid‐way through – so, be upfront with all relevant details, as all of this creates uncertainty for the patients involved…”* *“The rheumatologists who are doing clinical trials don't always tell all of their patients that they are doing the trials”* *“Also, need to explain to patients all the details of what the research is about; this helps with the trust issues.”*
	Dedicated research coordinator visits	*“Having someone from the community who is part of team, is involved [in] clinical trials, [and] can deliver the information honestly, not ambiguously, is important to maintain trust of the patients.”* *“Details on what a visit will look like can help…”*
	Being off therapy	*“… has brought up research opportunities, but in some cases, [I] would have had to come off my meds for 3 months…”* *“Back and forth on medicines that needed to be stopped.. Had abnormal PAP… But then PAP was normal but was on meds again… make sure your professionals are well trained in their roles so this doesn't happen…”*
Theme 2: Medical costs and insurance coverage	Financial resources	*“Doctors should tell patients about options like copay programs, if available…”* *“I try to find other 3rd party networks that can do administration of drugs.”*
	Insurance coverage and appeals	“*Patients are paying for things out of pocket if they have a high deductible…”* *“Insurance doesn't always want to pay for MRIs or CT scans… sometimes it is covered better if recommended by rheumatologists…”*
	Transparency of cost	*“Doctors must discuss ALL therapeutic options and their side effects with patients so patients can make their own care plans, especially in context of their insurance, costs, and more.”* *“I do agree, there should be cost transparency. However, sometimes the rheumatologist has limited time, so this doesn't happen as much as it could.”*
	Patient and provider advocacy	*“Tell the patient about options so they know they are there, and tell them you will advocate for them with the insurance company, if needed.”* *“Rheumatologists should try to help patients. Don't disregard or not recommend something just because the doctor assumes [their] insurer won't cover.”*

* Themes arranged from most common to least common listed frequency. Only key subcategories for each theme are shown. CT, computerized tomography; MRI, magnetic resonance imaging.

### Domain 1. Treatment and monitoring of LN and SLE (Table 2)

#### Theme 1: Medication side effects

The most common theme highlighted by Patient Panel members was the negative impact of treatment on quality of life. A patient taking mycophenolic acid stated that *“Food is a big part of [my] background and culture, and [I] love to cook, so having GI [stomach] issues [with medicine] really decreases [my] quality of life… this is important [to me]…”* Additionally, patients noted that balancing side effects with efficacy is important to them: *“Side effects of therapies do affect quality of life, but I am willing to take all of these side effects over kidney failure.”* Other subcategories that were emphasized included the impact of treatment on mental health (eg, depression, suicidal ideation, social withdrawal) and pill fatigue potentially resulting in nonadherence: *“I have pill fatigue, sometimes I just can't do it… It is mentally overwhelming resulting in pill fatigue…*”

#### Theme 2: Daily function

Maximizing daily function was a key priority for many patients. The ability to perform required or valued daily activities, such as cooking, being physically active with the family, were included under daily function by the patients. A patient stated, *“…My goal is to survive and have the best daily functioning as possible.”* Another patient quoted, “*Quality of life is my number 1 wish, but in reality, this doesn't seem possible.”* Next, patients prioritized avoiding flares and preventing organ damage to maintain their daily activities: *“Each flare has led to a major “failure” (eg, loss of sight, going to [the] hospital), so avoiding the flare is most important to me.”* An additional theme emerged regarding including patient preferences on the route of administration and number of medicines needed to maintain daily function, “*Mode of administration should be considered […] [Discuss] what is easier for the patient or preferred by them…”* Another patient mentioned, “*Start with one medicine, then add others, because then you have a better understanding of side effects and response to each one…”*

#### Theme 3: Treatment goals

Patients discussed that their main goal was survival, when it was in question. A patient said, *“First priority was survival at time of diagnosis because I was in [a] coma – so I just wanted to live.”* Additionally, patients highlighted that treatment goals should align with patient values and change based on disease severity and life phases, focusing on long‐term outcomes as disease progression allows. To elaborate, a patient stated, *“In the past, everything was failing, so some questions were more critical than others. When you are really sick, your goals are different. You can have other conversations when your illness slows down a little.”*

#### Theme 4: Monitoring and screening

Patients discussed the importance of frequent lab monitoring and how helpful it is to carefully monitor organ function and response to current therapy. They shared some concerns regarding undergoing invasive procedures (eg, kidney biopsy) but agreed that initial kidney biopsy for diagnosis was acceptable, assuming that proper sedation and anesthesia were administered: *“[I] had a kidney biopsy and was told that it would hopefully tell that [I] had LN…”* However, most patients were concerned about getting a repeat kidney biopsy: *“I would not likely want to do another biopsy because of other possible side effects like bleeding. I would only do it again if there was something going on with…labs or there was some other clear reason.”* Additionally, patients highlighted that receiving sedation did improve their overall experience of undergoing an invasive procedure like kidney biopsy compared to not receiving sedation or anesthesia. A patient mentioned that *“I was not sedated and it [kidney biopsy] was painful…”*

### Domain 2. Themes regarding clinical communication (Table 3)

#### Theme 1: Optimizing clinical communication

Patients suggested several strategies that could improve clinical care. A highly ranked strategy was supporting shared decision‐making during their disease course to foster trust and built strong relationships with patients. A patient quoted, “*Risks are very different to you over time, so doctors must continue to check in with their patients regularly over disease course to discuss risks vs. benefits.”* Another patient stated that “*Every side effect, severe or mild, should be discussed…This helps with decision‐making and being prepared*.” Additionally, offering a personalized, compassionate, and collaborative team‐based approach, such as including a pharmacist in the care team, was considered extremely valuable. A patient stated, “*My pharmacist also suggested where I could reduce certain medications, all of which was helpful and made things easier to manage.”* Another patient said, “*The best decision depends on the drug sometimes, and the trust the patient has in their doctor…health care team…”*

#### Theme 2: Provider and structural impediments to effective communication

Many patients reported experiencing a significant power differential in their interactions with health care providers, a dynamic that can be reinforced or even exacerbated by the language some providers use. Judgmental communication, for instance, negatively impacted patients’ mental health and resulted in mistrust. As one patient advised, “*Don't be judgmental, but be curious about why they [the patient] may decline certain treatment options.”* Another patient described the impact of such communication, stating, *“Being dismissive…makes [a] patient feel worse and less open or willing to listen to their doctor.”* Beyond individual interactions, broader negative communication patterns, such as experiencing structural racism, also fostered mistrust and negatively affected patients’ feelings and care. To elaborate, a patient stated, *“…patients are often dismissed, even more so for people of color and women.”*

### Domain 3. Improving transparency and information sharing (Table 4)

#### Theme 1: Clinical trial participation

Patients highlighted a need for building trust among communities to ensure broader clinical trial recruitment and representation from diverse communities. They recommended that providing more detailed information about the medication, placebo, inclusion criteria and research outcomes could help build trust. A patient reported, “*Companies should answer questions and explain things to the community to build trust. Things need to be translated to communities so you can get them to participate in the trials.”* Moreover, patients suggested that the involvement of patients in clinical trial design and engaging patient advocates as research coordinators or on research teams could help build trust and ensure broader representation. A patient stated, *“Having someone from the community who is part of team, is involved [in] clinical trials, [and] can deliver the information honestly, not ambiguously, is important to maintain trust of the patients.”* A suggestion was made to offer a visit with a research coordinator and culturally appropriate materials to reassure possible research participants and increase participation across communities. Finally, they shared concerns about discontinuing key therapies to enroll in clinical trials: “*…has brought up research opportunities, but in some cases, [I] would have had to come off my meds for 3 months…”*

#### Theme 2: Medical costs and insurance coverage

Beyond clear communication about their medical conditions and treatment options, patients strongly emphasized their need for practical assistance in navigating the complex landscape of health care costs and insurance coverage. Many expressed feeling overwhelmed or stressed with managing these financial aspects, highlighting it as a barrier to accessing and adhering to care, and had a negative impact on their mental health. For example, one patient articulated this need clearly, stating, *“Doctors must discuss ALL therapeutic options and their side effects with patients so patients can make their own care plans, especially in context of their insurance, costs, and more.”* Other patients supported this sentiment and expressed a desire for transparency and partnership, where clinicians actively help patients understand the financial impact of their health care choices. Without support, patients expressed they may face unexpected out‐of‐pocket expenses, struggle to afford prescribed medications or procedures, or even delay or forego necessary care, ultimately impacting their health outcomes and overall well‐being. One patient noted, *“Patients are paying for things out of pocket if they have a high deductible…”*

#### Aligning treatment guidelines and evidence‐based recommendations with patients’ priorities and values

During the Voting Panel discussions, following the presentation of the graded evidence for each potential recommendation, Patient Panel representatives were invited to share the key themes and patient perspectives that had emerged from the two Patient Panel meetings. Such discussions helped align guideline recommendations with patients’ preferences. For example, when discussing repeat kidney biopsy to monitor treatment response, the panel heard a strong patient preference to limit invasive procedures, or at least to have a dedicated discussion on the specific benefits of repeating the biopsy versus empiric treatment escalation. Giving primacy to patient preferences likely influenced the voting on guideline recommendations.

In several instances during the Voting Panel meetings, physicians and patients worked together to clarify recommendations on complex topics, such as glucocorticoid use, balancing treatment efficacy with side effects, tailoring treatment per individual patient priorities, life phase, and values. For example, there was substantial variability regarding glucocorticoid dosing and tapering among the physician Voting Panel members. A nuanced discussion between physicians and Patient Panel representatives highlighted the need to minimize glucocorticoid exposure over time to limit side effects. However, Patient Panel representatives also conveyed their willingness to accept higher doses of glucocorticoids to gain disease control, provided that the benefits and harms were thoroughly discussed and a clear tapering plan was established.

Next, discussions highlighted the importance of contextualizing therapeutic choices within patients' broader life circumstances, including life stage and family planning. The Voting Panel's guidance to favor mycophenolate over cyclophosphamide, or to use the Euro‐Lupus low‐dose cyclophosphamide regimen if cyclophosphamide was deemed necessary, directly addresses the significant concerns raised by both physicians and Patient Panel members regarding the potential for cyclophosphamide‐induced infertility. This approach moves beyond simply treating the primary condition (SLE or LN) and demonstrates a patient‐centered commitment to proactively mitigate long‐term impacts on reproductive health and future family planning desires. It's a clear example of how broader therapeutic strategies must thoughtfully weigh treatment efficacy alongside individual patient priorities and life goals during different stages of life (eg, during reproductive years), ensuring that counseling and treatment choices support not just immediate health needs but also long‐term quality of life and personal aspirations.

**Figure 1:**
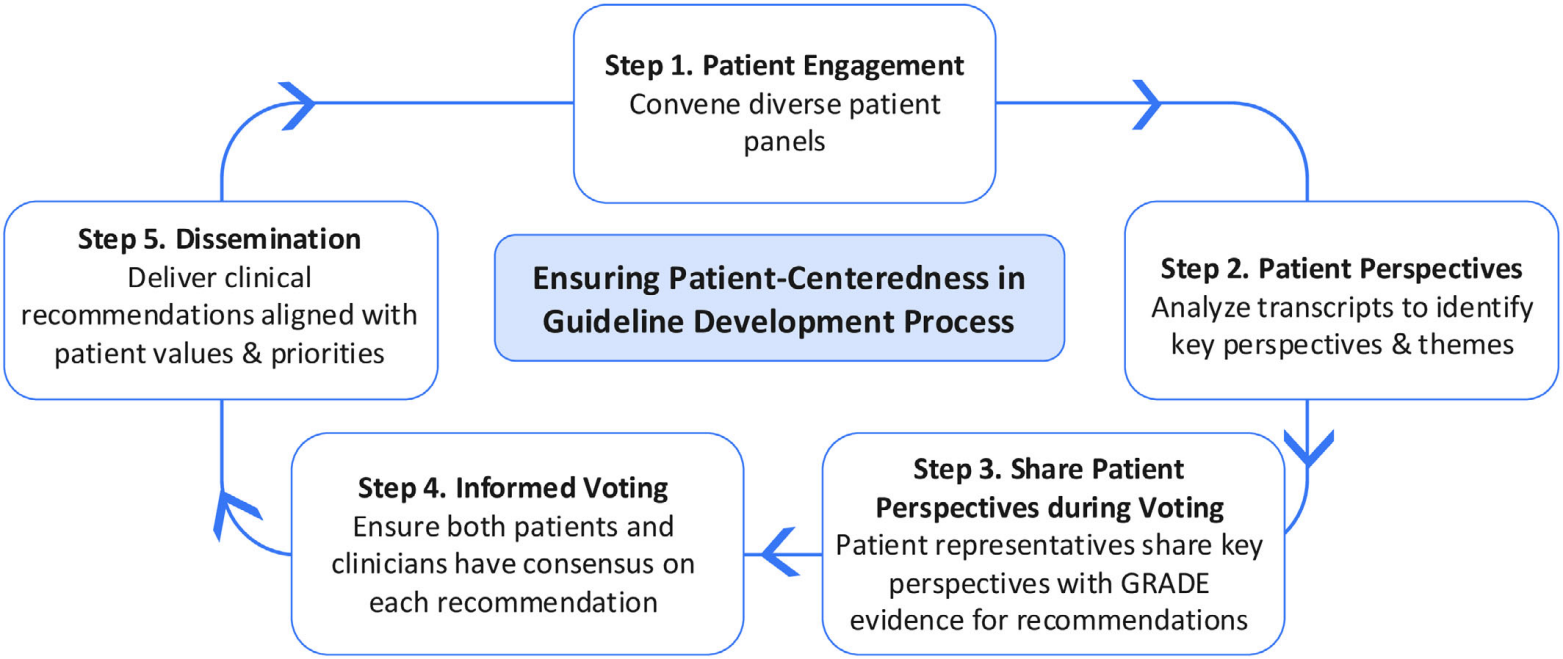
A framework to ensure the development of patient‐centered approach in clinical guideline development and potential improving clinical care.

The systematic approach that was adopted to align guideline recommendations with the Patient Panel's feedback informed our conceptual framework (Figure 1). This framework or approach can be used by other groups to develop clinical practice guidelines and potentially better align care with patient values and preferences.

## DISCUSSION

This study summarizes the processes employed to identify and incorporate patients’ experiences, perspectives, and priorities in the development of ACR LN and SLE screening, monitoring and treatment guidelines. These themes that arose from those discussions included input from Patient Panel members who participated on the Voting Panel, and were pivotal for aligning final guideline recommendations with patient perspectives, priorities, and values. Additionally, this work underscores the importance of tailoring treatment choices with patients' broader life circumstances, including life stage—recognizing that these will change over time so should be continually reassessed. Finally, integrating patient experiences helped broaden perspectives of the Voting Panel and develop treatment and management recommendations based on real‐world patient experiences, enhancing the clinical applicability of LN and SLE guideline recommendations.

Engaging patients in developing treatment and management guidelines is important.^34^ Several studies have explored patient experiences in SLE and LN.^10^, ^19^, ^35^, ^36^ Yet, this work was distinct as it was one of the first to directly integrate patient perspectives and patient members in the voting process to inform clinical guideline development aligned with real‐world patient experiences for SLE and LN.^37^, ^38^ Many previous efforts have documented patient priorities separately or retrospectively; our approach established a partnership where patient‐defined themes actively shaped recommendation discussions in real‐time. This work adapted an approach used by the 2023 ACR/CHEST ILD and other ACR guidelines to gather patient values and perspectives and integrate them into the Voting Panel decision‐making instead of only independently reviewing population, intervention, comparator, and outcomes (PICO) questions with the Patient Panel.^26^ This approach helped our team gather and share perspectives that spanned multiple PICO questions and clinical scenarios, including challenging and complex topics like family planning and treatment choices, personalized care, building trust, and effective, respectful communication. Moreover, the diversity of our Patient Panel, encompassing varied racial and ethnic backgrounds, socioeconomic statuses, and disease experiences and durations, was a strength. This heterogeneity likely contributed to the richness and breadth of themes identified, particularly concerning structural racism and health care access disparities, which may be less prominent in some cohorts.

The themes emerging from our panel resonate with much of the existing literature on patient experiences with chronic illness, including the significant burden of treatment side effects, the desire for shared decision‐making, and challenges with health care communication.^10^, ^19^, ^35^, ^36^ Particularly, patients highlighted the importance of survival as the key priority and tailoring treatment per individual patient preferences, life phases, and disease severity using a multidisciplinary, team‐based approach.^26^, ^39^, ^40^, ^41^ This highlights the importance of ongoing communication supported by shared decision‐making strategies to ensure that management is aligned to patients' evolving priorities based on disease severity and life phases.^20^, ^42^, ^43^ The emphasis on preserving organ function to prolong survival and improve quality of life while minimizing medication side effects reflected a desire of patients to support their overall well‐being and ensure better long‐term outcomes and survival. The explicit linkage of these patient‐articulated needs to the formulation of guideline recommendations distinguishes this study and provides a clear pathway for translating patient voices into actionable clinical guidance.

Additionally, effective communication and a strong patient‐physician relationship were noted as crucial elements of patient‐centered care. Further, cost and insurance issues were widely acknowledged, and our findings underscore the profound need for proactive, clinician‐guided navigation of these complexities, a theme that may be particularly pronounced in the US health care context represented by our panel.^44^, ^45^, ^46^, ^47^

Our study had a number of strengths including having a diverse group of Patient Panel participants, and the triangulation of data sources to enhance credibility and rigor. We also acknowledge some study limitations. First, although our Patient Panel included multiple perspectives, certainly not all possible perspectives were included. Second, it is important to consider that the experiences and challenges highlighted by patients, particularly those related to navigating care and costs, may be significantly influenced by the specific structure and complexities of the US health care system, and the regions of the United States with higher representation in the Patient Panels (eg, 47% of the Patient Panel members were from the South followed by 35% from the East). Consequently, some findings may have limited generalizability to patient experiences in countries with different health care models. Third, the data on the duration of disease (SLE or LN) and history of being on dialysis or undergoing a kidney transplantation were not collected. Therefore, some patient perspectives could have been missed in our analysis and could limit generalizability of our findings. Finally, significant differences in patient perspectives were not identified among patients with and without LN. However, it is important to note that majority of patients (77%) had LN. Therefore, future research should further explore these differences, particularly in patients without LN who have a less aggressive disease course and may be on less immunosuppression.

In conclusion, this study provides valuable insights into the experiences, perspectives, and priorities of patients with SLE and LN. The findings underscore the importance of patient‐centered care, effective communication, and addressing systemic barriers using multidisciplinary team‐based and patient‐centered approaches. By integrating patient perspectives into guideline development, we can continue towards a truly collaborative and patient‐centered approach for people with LN and SLE.

## AUTHOR CONTRIBUTIONS

All authors contributed to at least one of the following manuscript preparation roles: conceptualization AND/OR methodology, software, investigation, formal analysis, data curation, visualization, and validation AND drafting or reviewing/editing the final draft. As corresponding author, Dr Garg confirms that all authors have provided the final approval of the version to be published and takes responsibility for the affirmations regarding article submission (eg, not under consideration by another journal), the integrity of the data presented, and the statements regarding compliance with institutional review board/Declaration of Helsinki requirements.

## Supporting information


**Disclosure Form**:

**Supplementary File 1** A Priori Questions from Patient Panel Meetings

**Supplementary Table 1** Consolidated Criteria for Reporting Qualitative Research (COREQ): A 32‐Item Checklist
