# Supplementary material for: A Qualitative Analysis of Patient Perspectives and Preferences in Lupus Management to Guide Lupus Guidelines Development
Source: Arthritis Care Res (Hoboken). 2025 Nov 3;78(7):925–35. doi: 10.1002/acr.25693 (PMC13313109; doi:10.1002/acr.25693)
Supplement: Supplementary file 2 — Supplementary File 1 A Priori Questions from Patient Panel Meetings [file ACR-78-925-s001.docx]

**Supplementary File 1.** A Priori Questions from Patient Panel Meetings

**Section 1:** A Priori Questions- Meeting 1

**Focus:** Patient Perspectives on Lupus Nephritis (LN)

**Questions:**

**I. Questions Related to Treatment**

1. **When thinking about medications for lupus nephritis, how important are the following outcomes (benefits or harms of treatment)?**
   1. **Preservation of kidney function**—this means that kidneys work on their own without help from dialysis or need for transplantation.
   2. **Risk of flares of lupus nephritis**—flares are when the kidney disease becomes more active. During LN flares, there is increased risk of permanent damage to the kidney and risk of decreased kidney function.
   3. **End stage kidney disease**—this refers to permanent kidney damage needing either dialysis or a kidney transplant
   4. **Other side effects**
   5. **Risk of infections from the treatment**
   6. **Reducing the total dose of steroids** (e.g. prednisone or methylprednisolone) taken while on a treatment. The goal of some trials is to reduce the total dose of steroids.
   7. Ability to do your **daily required activities**
   8. Ability to participate in **activities for enjoyment**
   9. **Quality of life**
2. **How do you feel about the following side effects? Are some potential harms harder to accept? Would how severe your illness is** **make you more willing to accept potential harms?**
   1. Diarrhea, abdominal pain, nausea/vomiting or other side effects such as headache or fatigue
   2. An increased risk of infection
   3. An increased risk of developing cancer
   4. Potential effects on fertility
   5. ***Are there other potential harms that are important to consider?***
3. Using a combination of medications might be more effective but may have the potential to cause more side effects and usually means taking more pills per day. **What do you think about starting with one treatment vs starting with a combination of treatments? Would you prefer to start with one treatment and possibly add on other treatments, or would you prefer to start with multiple treatments and consider reducing the treatments if you show improvement?**
4. When considering medications, there are often different routes of administration such as tablets, injections you give yourself or infusions you receive at an infusion center. There are also different frequencies of medication administration. **How important is the route of administration of frequency of treatment to your decision making? Are there cases where you might choose a medication that is less likely to work based on different routes of administration?**
5. **Do you have any thoughts about some of the specific treatments being considered in these guidelines? Specifically:**
   1. **Cyclophosphamide (Cytoxan)**
      1. Oral treatment or monthly infusion that treats lupus nephritis but increase the risk of infection, may affect fertility, and carries a low risk of future cancers.
   2. **Mycophenolate (Cellcept)**
      1. Similar benefits for treating lupus nephritis as cyclophosphamide but has more diarrhea/GI side effects. There is also an increased risk of infection, but not as much as cyclophosphamide. Cellcept is usually 4-6 pills/capsules two times per day.
6. **Other thoughts about medications?**
   1. **Rituximab (Rituxan)**
      1. Infusion treatment (usually 2 doses every 6 months) with few physical side effects other than infusion reactions, but there is mixed evidence about effectiveness and potential risk of serious infections (including COVID).
   2. **Belimumab (Benlysta)**
      1. Either monthly infusion or weekly injections. Not many physical side effects but may decrease effectiveness of vaccines and, therefore, increase risk of infections.
   3. **Other oral immunosuppressants**, which may increase the risk of infection and may have other side effects including nausea or diarrhea: *Leflunomide (Arava),* *Methotrexate, Azathioprine (Imuran), Tacrolimus (Prograf), Cyclosporine or Voclosporine (Lupkynis)*
   4. **Steroids** (e.g. *prednisone, methylprednisolone/medrol)* which work quickly but can cause a range of side effects especially at higher doses including weight gain, diabetes, increased risk of infection and negative impact on bone health.
7. **Are there other important considerations when thinking about what medication to take?**

**IV. Questions Related to the Monitoring of Lupus Nephritis**

1. **Do you have thoughts, concerns, or experiences to share regarding how often blood and urine tests should be done to monitor lupus nephritis?**
2. Which of the following outcomes are important to you in regard to **benefits of monitoring for lupus nephritis:**
   1. Preservation of kidney function
   2. Flare of lupus nephritis
   3. End stage kidney disease (which means either dialysis or transplant)
   4. Reduction of total steroid dose
3. Do you have specific thoughts, concerns, or experiences to share about any of the **following tests used to monitor for lupus nephritis (e.g. cost, inconvenience, discomfort, risks):**
   1. Blood tests
   2. Urine tests
   3. Imaging (X-rays, MRIs, CTs)

**III. Questions Related to Kidney Biopsy**

1. What *concerns* would you have if your physician recommended a **kidney biopsy?**
2. **Would you agree to a kidney biopsy if recommended by your physician** or would you need further information?
3. Do you have specific thoughts, concerns, or experiences to share about **kidney biopsies**?

**IV. Questions Regarding Dialysis and Renal Transplant**

1. **If your physician recommended dialysis** (a process by which the blood is filtered via a catheter through a machine—dialysis is required at least several times per week once started) due to poor kidney function, **which of the following outcomes would be most important to you:**
   1. Quality of life
   2. Risk of lupus flare
   3. Improved survival
   4. Risk of infection
   5. Risk of cardiovascular disease (e.g. heart attacks, strokes)
2. **If your physician recommended a preemptive kidney transplant instead of starting dialysis, which of the following outcomes would be most important to you:**
   1. Quality of life
   2. Risk of lupus flare
   3. Improved survival
   4. Risk of infection
   5. Risk of cardiovascular disease (e.g. heart attacks, strokes)
3. **If your physician recommended dialysis/renal transplant preventatively, what are your thoughts?**

**Section 2:** A Priori Questions – Meeting 2

**Focus:** Patient Perspectives on Systemic Lupus Erythematosus (SLE)

**Questions:**

**I. Goals of Treatment**

1. We would like to learn about your ***goals of treatment*** as a person with lupus. We are interested in knowing what is most important to you. Below is a list of potential goals. We welcome any additions to this list that you suggest. **Please identify the three most important goals to you.**
   1. Prevent flares of disease
   2. Protect organ function (e.g. protecting joints from damage, skin from scarring)
   3. Avoid risk of infection from immunosuppressive medications
   4. Minimizing side effects (e.g. GI upset/diarrhea, headaches, etc)
      1. *Are there specific side effects that are most disruptive to you?*
   5. Maximizing quality of life
      1. *What does this mean to you? How can your healthcare provider support you in achieving your best quality of life?*
   6. Ability to do your daily required activities
   7. Ability to participate in activities for enjoyment
   8. Reducing steroid dosing (e.g. prednisone) to the lowest dose possible
   9. Do these priorities change if there are severe manifestations of lupus present (e.g. when it affects the brain or spinal cord/neuropsychiatric lupus)?
   10. Other factors not listed?

II. **Questions Related to Treatment**

1. **How do you feel about the following side effects? Are some potential harms harder to accept?** **Would how severe your illness is make you more willing to accept potential harms?**
   1. Diarrhea, abdominal pain, nausea/vomiting or other side effects such as headache or fatigue
   2. An increased risk of infection
   3. An increased risk of developing cancer
   4. Potential effects on fertility
   5. *Are there other potential harms that are important to consider?*
2. **What are your preferences for tapering medications** (decreasing the dose slowly over time until it is stopped)? **Do you have concerns about slow tapers?**
3. When considering medications, there are often different routes of administrations, such as tablets, joint injections, skin injections, injections you give yourself or infusions you receive at an infusion center. There are also different frequencies of medication administration. **How important is the route of administration or frequency of treatment to your decision making?** **Are there cases where you might choose a medication that is less likely to work based on different routes of administration or other use scenarios?**
4. **Are there other important considerations when thinking about what medication to take?**
5. We recognize that taking many medications for long periods of time can be difficult, which may lead to missing doses. **Do you have any suggestions as to how your healthcare provider could raise this issue to discuss the challenges?** Are there things **healthcare providers should know about adherence** (e.g. ability to stay on a prescribed set of medicines) from a patient point of view?
6. Using a combination of medications might be more effective but may have the potential to cause more side effects and usually means taking more pills per day. **How do you think about starting with one treatment vs. starting with a combination of treatments**? **Would you prefer to start with one treatment and possibly add on other treatments, or would you prefer to start with multiple treatments and consider reducing the treatments if you show improvement?**
7. **Do you have any thoughts about some of the specific treatments being considered in the guidelines? Specifically:**
   1. **Steroids (e.g. prednisone, methylprednisolone/medrol)** which work quickly but can case a range of side effects especially at higher doses including weight gain, diabetes, increased risk of infection and negative impact on bone health.
   2. **Belimumab (Benlysta)**
      1. Either monthly infusions or weekly injections. Not many physical side effects but may decrease effectiveness of vaccines and, therefore, increase risk of infection.
   3. **Anifrolumab (Saphnelo)**
      1. Monthly infusion that targets a single protein. This is a newly approved medication for lupus and has been helpful in reducing disease activity. Side effects include increased risk for infection, specifically shingles (herpes zoster).
   4. **Cyclophosphamide (Cytoxan)**
      1. Oral treatment or monthly infusions that treat lupus but increase the risk of infection, may affect fertility, and carries a low risk of future cancers.
   5. **Mycophenolate (Cellcept)**
      1. Benefits for treating certain lupus manifestations
      2. Common diarrhea/GI side effects. There is also an increased risk of infection, but not as much as cyclophosphamide. Cellcept is usually 4-6 pills/capsules two times per day.
   6. **Rituximab (Rituxan)**
      1. Infusion treatment (usually 2 doses every 6 months) with few physical side effects other than infusion reactions, but there is mixed evidence about effectiveness and potential risk of serious infections (including COVID).
   7. **Other oral immunosuppressants** which may increase the risk of infection and may have other side effects including nausea or diarrhea: *Leflunomide (Arava), Methotrexate, Azathioprine (Imuran), Tacrolimus (Prograf), Cyclosporine or Voclosporine (Lupkynis)*

**III. Questions Related to Monitoring Lupus**

1. Do you have thoughts, concerns, or experiences to share regarding ***how often* doctor visits, blood and urine tests should be done to monitor lupus?**
2. **Which of the following outcomes are important to you in regard to *benefits of monitoring lupus*:**
   1. Preservation of organ function
   2. Flare of lupus
   3. Reduction of total steroid dose
3. **Do you have specific thoughts, concerns, or experiences to share about any of the following tests used to monitor for lupus (e.g. cost, inconvenience, discomfort, risks, unclear what these tests measure):**
   1. Blood tests
   2. Urine tests
   3. Imaging (X-rays, MRIs, CTs)

**IV. Questions Related to Research Participation**

1. We expect that there will be more research trials for new medicines in lupus. Research trials can be an important way to test a medicine to see if it works well and what the side effects are. **Do you have concerns about research participation? What would you need to know before consenting to participate in a study?**

**V. Other Comments?**
