# Supplementary material for: A Qualitative Analysis of Patient Perspectives and Preferences in Lupus Management to Guide Lupus Guidelines Development
Source: Arthritis Care Res (Hoboken). 2025 Nov 3;78(7):925–35. doi: 10.1002/acr.25693 (PMC13313109; doi:10.1002/acr.25693)
Supplement: Supplementary file 3 — Supplementary Table 1 Consolidated Criteria for Reporting Qualitative Research (COREQ): A 32‐Item Checklist [file ACR-78-925-s003.docx]

**Supplementary Table 1**. Consolidated Criteria for Reporting Qualitative Research (COREQ): A 32-Item Checklist

| **Item** | **Guide Questions/Description** | **Response** |
| --- | --- | --- |
| **Domain 1: Research Team and Reflexivity** | | |
| **Personal Characteristics** | | |
| 1. Interviewer/ Facilitator | Which author/s conducted the interview or focus group? | Shivani Garg, Linda Hiraki, and Mary Beth Son supported by two ACR staff members (Amy Turner and Regina Parker) |
| 2. Credentials | What were the researcher's credentials? *E.g. PhD, MD* | Shivani Garg MD PhD, Linda Hiraki MD, ScD, Mary Beth Son MD, Amy Turner |
| 3. Occupation | What was their occupation at the time of the study? | SG: Medical Director of UW Lupus and Lupus Nephritis Clinics; Clinical Rheumatologist; Assistant Professor of Medicine, Rheumatology  LH: Staff Physician, Division of Rheumatology, Department of pediatrics, The Hospital for Sick Children, Toronto, ON, Canada; Clinician-Scientist, Genetics & Genome Biology MBS: Pediatric rheumatologist at Boston Children's Hospital; Clinical Chief, Division of Immunology; Director, Services and Outreach; Program Director, Rheumatology Program; Associate Professor of Pediatrics, Harvard Medical School  AST: American College of Rheumatology Senior Director, Quality |
| 4. Gender | Was the researcher male or female? | All participating researchers are female |
| 5. Experience and Training | What experience or training did the researcher have? | SG: Medical Director of UW Lupus and Lupus Nephritis Clinics; Clinical Rheumatologist; Assistant Professor of Medicine, Rheumatology LH: Staff Physician, Division of Rheumatology, Department of pediatrics, The Hospital for Sick Children, Toronto, ON, Canada; Clinician-Scientist, Genetics & Genome Biology MBS: Pediatric rheumatologist at Boston Children's Hospital; Clinical Chief, Division of Immunology; Director, Services and Outreach; Program Director, Rheumatology Program; Associate Professor of Pediatrics, Harvard Medical School  AST: American College of Rheumatology Senior Director, Quality |
| **Relationship with Participants** | | |
| 6. Relationship Established | Was a relationship established prior to study commencement? | Yes, patients received an evidence report synopsis and participated in an orientation webinar meant to provide information and guidance on interpreting the evidence report prior to the Patient Panel meeting. |
| 7. Participant Knowledge of the Interviewer | What did the participants know about the researcher? *e.g. personal goals, reasons for doing the research* | Participants and interviewers introduced themselves at the beginning of both Patients Panels on a video platform. The goals of the Patient Panels, namely, to elicit patient perspectives and priorities and to include them in guideline development, were reviewed at those times. |
| 8. Interviewer Characteristics | What characteristics were reported about the interviewer/facilitator? *e.g. Bias, assumptions, reasons and interests in the research topic* | No characteristics were specifically reported about the interviewer/facilitator. |
| **Domain 2: Study Design** | | |
| **Theoretical Framework** | | |
| 9. Methodological Orientation and Theory | What methodological orientation was stated to underpin the study? *e.g. grounded theory, discourse analysis, ethnography, phenomenology, content analysis* | Content analysis |
| **Participant Selection** | | |
| 10. Sampling | How were participants selected? *e.g. purposive, convenience, consecutive, snowball* | Participants were selected using national surveys, recommendations from the ACR LN and SLE guideline development group members, and previous ACR patient panel/volunteer lists. Participants for the focus groups were purposefully recruited to represent different cultural, social, and ethnic backgrounds, disease duration, and age of onset, to capture different patient perspectives and values. |
| 11. Method of Approach | How were participants approached? *e.g. face-to-face, telephone, mail, email* | Participants were approached using email. |
| 12. Sample Size | How many participants were in the study? | 19 individuals with LN and/or SLE diagnoses participated in the ACR LN and SLE Guidelines Patient Panels. |
| 13. Non-Participation | How many people refused to participate or dropped out? Reasons? | Among 19 patient panel members who attended one or both patient panel meetings, 17 members gave verbal consent to use their feedback shared during Patient Panel meetings for the qualitative analysis. 2 of the participants could not be reached, so verbal consent was not explicitly given, and their feedback was not incorporated into the qualitative analysis. |
| **Setting** | | |
| 14. Setting of Data Collection | Where was the data collected? *e.g. home, clinic, workplace* | Two four-hour Patient Panel meetings were held virtually on a secure, video-call platform. |
| 15. Presence of Non-Participants | Was anyone else present besides the participants and researchers? | Only researchers and patients were present, including supporting ACR staff members experienced with ACR guideline development and processes (AST and RP). |
| 16. Description of Sample | What are the important characteristics of the sample? *e.g. demographic data, date* | Fifteen patients participated in the LN Patient Panel meeting and 13 in the SLE patient panel meeting; 9 patients participated in both panel meetings. A total of 19 unique individuals participated on either patient panel, and among these 17 patients provided consent to use their feedback for analysis and inclusion in aggregate data for this paper. 88% were females, 55% were of Black race, and 77% had LN. The majority of patient panel members were from the South (45%) followed by the East (35%) |
| **Data Collection** | | |
| 17. Interview Guide | Were questions, prompts, guides provided by the authors? Was it pilot tested? | Yes, questions, prompts, and guides are included in Supplementary File 1 by the authors. A semi-structured outline of topics covered by the Population, Intervention, Comparator, Outcome (PICO) questions and literature review was shared with participants prior to the meetings as well as utilized in the Patient Panel meetings. |
| 18. Repeat Interviews | Were repeat interviews carried out? If yes, how many? | No, repeat interviews were not carried out. Only the two Patient Panel meetings were conducted. |
| 19. Audio/Visual Recording | Did the research use audio or visual recording to collect the data? | The Patient Panel meetings were audio recorded by the ACR team (AST and RP), and quotes and comments made by Patient Panel members were documented. |
| 20. Field Notes | Were field notes made during and/or after the interview or focus group? | Yes, quotes and comments made by Patient Panel members were documented. Key themes were noted by each facilitator. |
| 21. Duration | What was the duration of the interviews or focus group? | Each focus group lasted four hours. |
| 22. Data Saturation | Was data saturation discussed? | Yes, adjustments to the coding scheme were made iteratively between each reading of transcripts until thematic saturation was reached. |
| 23. Transcripts Returned | Were transcripts returned to participants for comment and/or correction? | Text transcripts were independently reviewed by the facilitators for accuracy and to achieve immersion. Key themes and perspectives identified were shared with patient panel members. |
| **Domain 3: Analysis and Findings** | | |
| **Data Analysis** | | |
| 24. Number of Data Coders | How many data coders coded the data? | Content analysis was performed by two independent reviewers (including 1 facilitator, SG, and 1 non-facilitator, LH). |
| 25. Description of the Coding Tree | Did authors provide a description of the coding tree? | Yes, themes and subthemes were thoroughly discussed and summarized in tables. |
| 26. Derivation of Themes | Were themes identified in advance or derived from the data? | Key themes were generated from the data, which informed the coding scheme for the content analysis. |
| 27. Software | What software, if applicable, was used to manage the data? | Transcripts were coded and managed using NVivo software. |
| 28. Participant Checking | Did participants provide feedback on the findings? | Yes, key themes were reviewed with the Patient Panel members to obtain feedback on the findings. |
| **Reporting** | | |
| 29. Quotations Presented | Were participant quotations presented to illustrate the themes/findings? Was each quotation identified? *e.g., participant number* | 1-3 participant quotations were presented to supplement each theme/finding described in the paper. All quotes are unidentified, but context is given when necessary. |
| 30. Data and Findings Consistent | Was there consistency between the data presented and the findings? | Yes, the presented data supported the integration of patient experiences and findings in the clinical practice guideline development process and aligned management recommendations to real-world patient experiences and priorities, thereby enhancing the clinical applicability of the ACR LN and SLE guidelines. |
| 31. Clarity of Major Themes | Were major themes clearly presented in the findings? | Yes, thematic analysis revealed nine patient-reported key themes across three domains. |
| 32. Clarity of Minor Themes | Is there a description of diverse cases or discussion of minor themes? | Yes, our study highlights strategies to engage patients with diverse experiences to inform guideline recommendations per unique patient perspectives. Each theme discussed incorporates diverse cases and nuanced feedback. |
